# MRI-Based Inner Ear Assessment and Cochlin Tomoprotein-Based Evaluation of Perilymphatic Fistula in Patients with Sudden Hearing Loss

**DOI:** 10.3390/brainsci14070681

**Published:** 2024-07-05

**Authors:** Rayoung Kim, Denis Mihailovic, Conrad Riemann, Alexander Kilgué, Christoph Joachim Pfeiffer, Hans-Björn Gehl, Lars-Uwe Scholtz, Ingo Todt

**Affiliations:** 1 Department of Otolaryngology, Head and Neck Surgery, Medical Faculty OWL, Bielefeld University, Campus Klinikum Bielefeld, Teutoburgerstr. 50, 33604 Bielefeld, Germany; conrad.riemann@klinikumbielefeld.de (C.R.); alexander.kilgue@klinikumbielefeld.de (A.K.); christoph.pfeiffer@klinikumbielefeld.de (C.J.P.); lars-uwe.scholtz@klinikumbielefeld.de (L.-U.S.); todt@gmx.net (I.T.); 2 Department of Radiology, Medical Faculty OWL, Bielefeld University, Campus Klinikum Bielefeld, Teutoburgerstr. 50, 33604 Bielefeld, Germany; denis.mihailovic@klinikumbielefeld.de (D.M.);

**Keywords:** magnetic resonance imaging, 4 h delayed intravenous gadolinium-enhanced 3D FLAIR MRI, cochlin tomoprotein, sudden hearing loss, perilymphatic fistula

## Abstract

Objectives: To study the correlation between positive cochlin tomoprotein testing (CTP), magnetic resonance (MR) imaging, and the auditory and vestibular function amongst patients with sudden hearing loss. Study Design: Prospective case series. Methods: We prospectively examined eight patients who presented with sudden hearing loss (>60 dB) with or without vertigo or tinnitus. We performed an ELISA-based CTP detection test using middle ear lavage samples. In addition to the CTP examination, a magnetic resonance imaging (MRI) examination was performed using different sequences (T1 and a T1 sequence with a contrast medium (CM), a T2 sequence, 4 h delayed intravenous gadolinium-enhanced three-dimensional fluid-attenuated inversion recovery (3D FLAIR)). Results: All patients with sudden hearing loss (>60 dB) presented a non-specific contrast enhancement in the cochlea and vestibulum on the affected side on delayed 3D-FLAIR MRI. Four patients had a positive CTP test, suggesting a perilymphatic fistula (PLF). However, no specific MRI signal for a PLF was observed. Conclusions: Using multimodal diagnostic measures, such as CTP testing and different MRI sequences, no correlation could be found in patients with a PLF.

## 1. Introduction

A perilymphatic fistula (PLF) is an abnormal communication between the perilymph-filled inner ear and its outer surrounding spaces such as the middle ear, mastoid, or intracranial cavity [1]. Commonly, the leakage caused by a PLF occurs in the area of the round or oval window of the cochlea [2]. Trauma, idiopathic, or iatrogenic aspects after middle ear surgery, such as stapedotomy, are suggested as the etiological factors of PLFs. It is known that a PLF could cause variable clinical symptoms, such as sudden hearing loss, dizziness, impaired balance, and tinnitus [2,3]. Those symptoms, however, mimic the presentation of other inner ear pathologies, such as Ménière’s disease (MD), endolymphatic hydrops (EH), semicircular canal dehiscence (SCD), vestibular atelectasis, or vestibular migraine [1], so that the diagnostic tools to differentiate a PLF from those other pathologies is required.

The various clinical tests, including audiometric tests, vestibular evoked myogenic potentials (VEMPs), fistula tests, or caloric tests, may aid in determining the side of the affected ear during the assessment of a PLF [1]. However, those testing methods are restricted to distinguish PLFs from other causes of symptoms [4]. So far, an explorative tympanostomy is regarded as a gold standard for diagnosing a PLF. By observing the leakage of perilymph, a PLF is confirmed, and at the same time, a therapeutic option is offered to seal the round (RW) and oval window (OW). However, this diagnostic approach is subjective and implies the possibility of making a false positive diagnosis [5].

As a biomarker for diagnosis of a PLF, beta-trace protein, beta-transferrin, and CTP (cochlin tomoprotein) are suggested [3,6,7,8]. While ß-trace protein and ß-transferrin have been mentioned as possible diagnostic markers, both markers showed a low sensitivity for the presence of a PLF [3]. CTP, on the other hand, is an inner ear-specific protein used as a biochemical marker for PLF [6,7]. Through the CTP test with 98.2% specificity, a PLF could be diagnosed as distinguishable from other ear pathologies [8]. Currently, ELISA testing is time consuming, and the harvesting of a middle ear lavage (MEL) for CTP testing is based on a tympanotomy. Therefore, other techniques to support the diagnosis of a PLF are favorable.

Inner ear-specific aspects can also be assessed by CT or MRI examinations, particularly with 4 h delayed intravenous gadolinium-enhanced MRI sequences [9]. This validated technique allows for the visual evaluation of endolymphatic space after the contrast medium accumulates within the perilymphatic compartment via the blood–labyrinth barrier [10].

The morphological assessment of MRI was performed to identify the pathologies of the labyrinth, and the characteristics of images depending on pathology were suggested. Previously, several study groups suggested that a round window signal(RWS) on delayed 3D-FLAIR sequences is a sensitive sign of a PLF [11,12,13]. Another group suggested that a PLF could be diagnosed by investigating a fluid signal in the RW or the oval window (OW) niches in the CT and MRI (T2W without contrast) [13]. However, it must be mentioned that these studies are based on single-case observations or clinical assumptions without objectively determining a PLF. Furthermore, Eliezer et al. presented patients with sudden hearing loss and delayed 3D-FLAIR MRIs in their study, which showed no visible saccule (NVS). Two patients had a surgically confirmed PLF. They hypothesized that an NVS might be caused by a saccular rupture based on a PLF [14]. Unlike other causes of sudden hearing loss, a PLF is usually surgically treated by sealing the RW and OW. However, the appropriate treatment of a PLF is optimal if the diagnosis is performed precisely in time [1]. Otherwise, a pre-operative diagnosis of a PLF could enable us to avoid an unnecessary surgical approach.

We raised the hypothesis that if a PLF exists, the perilymphatic collapse on delayed 3D-FLAIR might be observed, causing an endolymph/perilymph shift. This study aims to analyze multimodal diagnostic measures with CTP examination and MRI scans to detect correlations in patients with sudden hearing loss.

## 2. Material and Methods

### 2.1. Subjects

In our prospective study, we evaluated eight patients (68.3 y mean age, six males, two females) presented in our hospital’s emergency unit with sudden sensorineural hearing loss between 2021 and 2022. Before a tympanostomy, steroid treatment was performed (10 days of steroids in a decreasing dose starting at 250 mg prednisolone) as a first-line treatment. If the mean bone conduction in pure tone audiometry after this first-line treatment was found to be 60 dB or higher, a tympanostomy with a window coverage was performed by two surgeons. MRI and CT pre-OP excluded other reasons for HL (e.g., schwannoma, meningioma).

### 2.2. Surgical Approach

The surgical coverage of the RW and OW was performed if a conservative treatment was not effective. An endaural access was performed, and the tympanic membrane (TM) was elevated. The middle ear was inspected and classified as a suspicious PLF. This was followed by rinsing the middle ear three times and taking the MEL. The RW and OW were covered with fascia, and the middle ear was filled with fibrin glue. Finally, the TM was repositioned and stabilized with silicone sheeting (Spiggle& Theis Medizintechnik GmbH, Overath, Germany) and a gelatin sponge (Curaspon^®^, Curamedical, Assendelft, The Netherlands).

### 2.3. CTP

The sample collection to detect CTP was performed by lavaging the middle ear cavity with 0.3 mL of saline and recovering the fluid, defined as the MEL. The collected MEL was centrifuged at 6000 rpm for 15 s (Eppendorf Systems, Hamburg, Germany), and the supernatant was collected for testing ELISA. The cut-off values for monoclonal AB ELISA CTP were as follows: <30 ng/mL was no PLF, 30–60 ng/mL was intermediate PLF, and >60 ng/mL was sure for PLF (CTP ELISA: TECAN/IBL:301170068 at an external laboratory in a blinded fashion).

### 2.4. Imaging

MRI examinations were performed on a 3-tesla MRI system (Ingenia, Philips, Best, The Netherlands) with a 16-channel array head coil. All patients underwent an MRI in different sequences, such as a T1 sequence with and without CM, a T2 sequence, an MRI examination 4 h after an intravenous application of Gadobutrol at a dose of 0.2 mL/kg. Three-dimensional-FLAIR was performed with the following protocol (time of repetition: 6000 ms, time of echo: 177 ms, time of inversion: 2000 ms, matrix size: 240,217, field of view: 190 mm, slice thickness: 0.8 mm, acquisition time: 12 min). Endolymphatic and perilymphatic spaces in the affected, and contralateral ears were compared with visual evaluation on MRI. Otherwise, we used the grading system of Bernaerts et al. for the diagnosis of EH [9].

Additionally, a CBCT (NewTom VGI, Verona, Italy) or multislice computed tomography (MSCT) 80 was carried out to identify the pathology and bony representation in all cases. CBCT parameters were as follows. FOV 15 × 15 cm, 10.48 mAS-20.52 mAS, KV 110, and 360° followed by 2D and 3D reconstructions at an external workstation (NNT, main station). The MSCT Toshiba Aquilion 80 protocol was as follows: slice thickness 0.5 mm, KV 120, MA 200, and rot. time 0.75.

### 2.5. Audiometric Tests

The pure-tone audiograms were evaluated per air and bone conduction at 250 Hz, 500 Hz, 1000 Hz, 2000 Hz, 3000 Hz, 4000 Hz, and 6000 Hz (AT1000, Auritec, Hamburg, Germany). The pure-tone average (PTA) was assessed as a mean value of the tonal threshold at the aforementioned seven frequencies.

### 2.6. Caloric Test

Caloric tests were performed by irrigating the ears with bithermal water at 30 °C and 44 °C for 40 s alternatively (ATMOS Variotherm plus; ATMOS MedizinTechnik, Lenzkirch, Germany). The induced nystagmus was recorded with video-oculography (Otometrics; Natus medical GmbH, Madison, WI, USA). Labyrinthic paresis was defined as a response difference of 20% or more between both ears [15].

### 2.7. Video Head Impulse Test (VHIT)

We performed the VHIT (Otometrics; Natus medical GmbH, Madison, WI, USA) with at least ten head impulses in the axis of each semicircular canal. The threshold for normal vestibulo-ocular reflex (VOR) gain was 0.7 for vertical canals and 0.8 for horizontal canals.

## 3. Results

The clinical findings are summarized in Table 1 and Table 2. In all patients with spontaneous hearing loss (>60 dB) included in this study, the delayed 3D-FLAIR MRI scan showed unspecific increased contrast enhancement in the area of the vestibulum, cochlea, and fundus of the internal auditory canal of the affected side. However, a RWS was not observed in any of the affected patients. A positive CTP result was detected in four patients, suggesting a PLF. We could observe the unspecific MR enhancement in the cochlea and vestibulum on delayed 3D-FLAIR MRI in patients with a positive CTP value, but no specific characteristic was found. In two patients, an inversion of the saccule to utricle area ratio (SURI) was observed on delayed 3D-FLAIR MRI, suggesting endolymphatic hydrops [16]; one of these patients had a high value of CTP (s. case 3). The other did not have a high CTP value (s. case 8). Two patients (in cases 5 and 8) with dehiscence of the superior semicircular canal (SSCD) were detected on computer tomography. In a delayed 3D-FLAIR sequence, the SSCD could be detected with a hyperdense sign in one of those patients (case 5) but not in the other patient. In case 2, a hyperdense sign is shown on the T1 sequence with and without a contrast medium, but there was a hypodense sign in the basal cochlea on the T2 sequence, suggesting that labyrinthitis is the acute symptom process being considered.

The mean PTA threshold of the affected side in all patients was 104.8 ± 9.7 dB (*n* = 8). The mean PTA threshold of the unaffected side was 42 ± 35.5 dB. The mean PTA threshold of the affected ear in four patients with PLF was 105.5 ± 9 dB. In the 3-week post-operative tone audiogram after the explorative tympanotomy with RW/OW sealing, the mean PTA level partially recovered from about 29.3 dB to 76.2 ± 28 dB in the patient with PLF. The regaining of the mean PTA level, about 14.3 dB from 104.1 ± 11.8 dB to 89.3 dB, could also be observed in patients without PLF (CTP negative) after the same surgical treatment.

Out of eight patients, 50% of the patients in this study showed a positive CTP value (>60 ng/mL), suggesting a PLF. All four patients with a positive CTP value had no vertigo subjectively. However, in case 1, we could observe a pathological gain (0.00) in the VOR test and a hyporeflexia on the affected ear in the caloric test.

In seven of the eight cases, intraoperatively, a track of PLF could be observed, but only four patients had a CTP-verified PLF. In case 4, no track of a PLF was observed intraoperatively, but the CTP value was high. There were no complications of surgery observed. None of these patients had a family history of otological deficit or exposure to chemotherapy containing platinum or aminoglycosides.

### 3.1. Case 1

**Figure 1:**
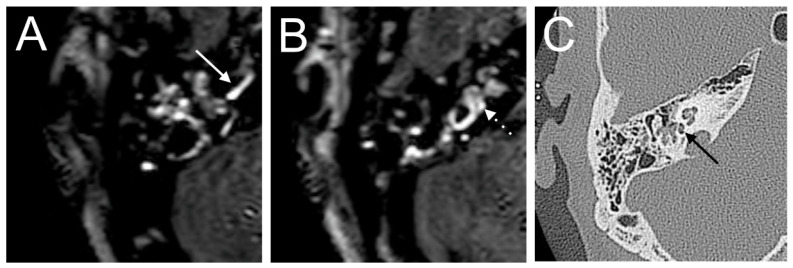
A 60-year-old male presented with a sudden hearing loss on the right side for one week. The axial 4 h delayed 3D FLAIR sequence shows a contrast enhancement in the cochlear basal turn (arrow) (**A**) and vestibulum (**B**). About 3/3 of fluid filling in the RW niche was observed (arrow, (**C**)).

A 60-year-old male presented with a sudden hearing loss on the right side for one week. The PTA was 110 dB on the right side. The patient had already had a sudden hearing loss on the same side in history, which was recovered. He denied having any other symptoms, such as tinnitus, vertigo, or aural fullness. He already had a cochlear implant on the left side related to sensorineural hearing loss. The visual examination showed a spontaneous nystagmus to the left side. A right-side hyporeflexia was observed in the caloric test. The VHIT test revealed a decreased right anterior (0.00) VOR gain. Intraoperatively, a PLF track around the RW niche was observed. The CTP test on the right side was positive, suggesting a PLF. On the 4 h delayed 3D-FLAIR image, a cochlear and vestibular contrast enhancement on the side of hearing loss was found (Figure 1). However, about 3/3 of fluid filling in the RW niche was observed in the affected ear in the CT, which could not be confirmed on T2W MRI. Additionally, the mesotympanon, hypotympanon, and mastoid were filled with fluid on the CT. Because of artifacts on the left side after CI, a side-to-side comparison was not possible. 

### 3.2. Case 2

**Figure 2:**
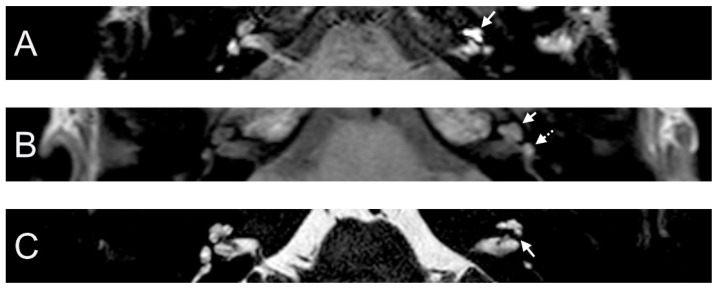
A 38-year-old female presented with sudden hearing loss on the left side and acute rotatory vertigo. The axial 4 h delayed 3D FLAIR sequence shows a contrast enhancement in the left cochlea (**A**). A cochlear (arrow) and vestibular (dashed arrow) contrast enhancement was observed in the axial T1 sequence (**B**). Hypointense signal intensity in the basal turn of the left cochlea was found on axial T2W (**C**).

A 38-year-old female presented with a sudden hearing loss on the left side with acute rotatory vertigo without tinnitus. The visual examination showed a spontaneous nystagmus to the right side. The audiogram revealed a PTA of 110 dB on the left. Intraoperatively, a PLF track around the RW niche was observed, but the CTP on the left side was negative. Hyperintense signal intensity was found on T1- and CE-T1-weighted MRI sequences. On the T2 sequence, a hypointense signal in cochlear basal turn was found on the affected side in comparison to the contralateral side. In consideration of T1 and T2 sequences and acute symptom development, we assumed that labyrinthitis caused the sudden hearing loss in this case. On the 4 h delayed 3D-FLAIR image, a contrast enhancement was found in the cochlea, vestibulum, and fundus on the side of hearing loss (Figure 2).

### 3.3. Case 3

**Figure 3:**
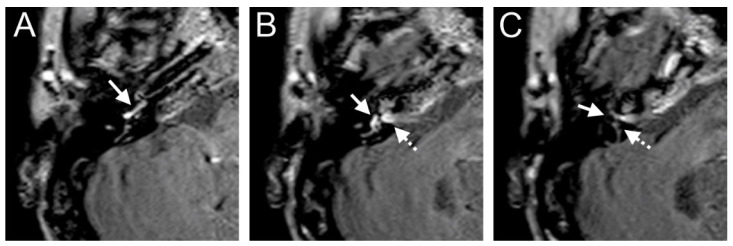
A 48-year-old male presented with a sudden hearing loss on the right side with tinnitus. The axial 4 h delayed 3D FLAIR sequence shows a contrast enhancement in the cochlea basal turn on the right side (**A**). In the same MR sequence, a vestibulum (arrow (**B**)) and fundus (dashed arrow (**B**)) and MR enhancement were observed, as well as an inversion of the saccule (arrow (**C**)) and utricle (dashed arrow (**C**)) area ratio.

A 48-year-old male presented with a sudden hearing loss on the right side with tinnitus. He showed no vestibular symptoms. The PTA was 110 dB on the right side. Intraoperatively, a PLF track around the RW niche was observed, and the CTP on the right side was positive. A normalized signal intensity of the vestibulum and cochlear was observed on T1W and CE-T1W. A hyperintense signal intensity was found on T2-weighted MRI sequences. On the 4 h delayed 3D-FLAIR image, a contrast enhancement was found in the cochlea, vestibulum, and fundus on the right side of the ear with hearing loss (Figure 3A,B). An RWS was not detected in the affected ear. In this MR sequence, we could also observe a SURI on the right side (vestibular endolymphatic hydrops, grade I) (Figure 3C).

### 3.4. Case 4

**Figure 4:**
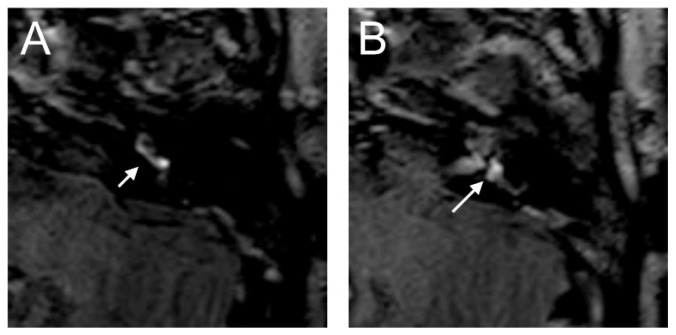
An 86-year-old male presented with a sudden hearing loss on the left side without vertigo and tinnitus. On the axial 4 h delayed 3D FLAIR sequence, there was a contrast enhancement in the cochlea basal turn (arrow (**A**)) and vestibulum (arrow (**B**)) on the left side.

An 86-year-old male presented with a sudden hearing loss on the left side without vertigo and tinnitus. The PTA was 110 dB on the left side, and there was a gradually progressive sensorineural hearing loss at high frequencies on the right side. No clinical sign of a PLF was observed intraoperatively, but CTP testing on the left side was positive. The contrast enhancement of vestibulum and cochlear basal turn was detected on the 4 h delayed 3D-FLAIR image (Figure 4). Otherwise, a hypointense sign was observed on the T1 sequence. A hyperintense signal intensity was found on the T2-weighted MRI in comparison to the other side.

### 3.5. Case 5

**Figure 5:**
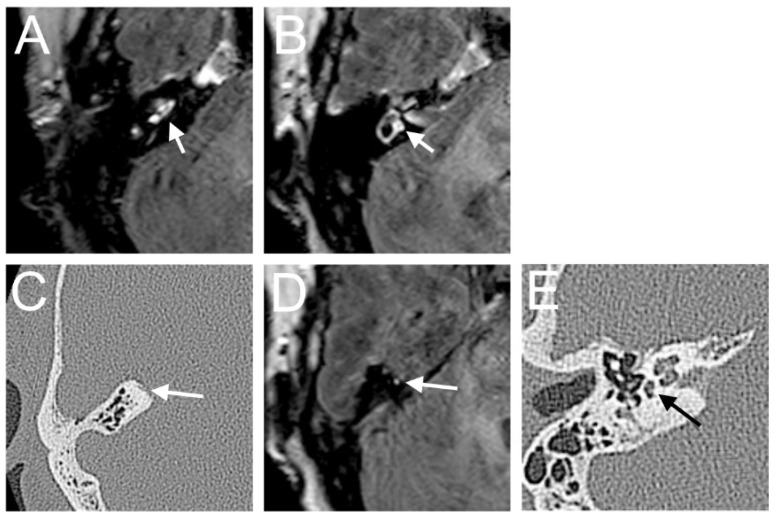
A 77-year-old patient with sudden hearing loss on the right side with rotatory vertigo. The contrast enhancement was seen in the right cochlea (**A**) and vestibulum (**B**) on the axial delayed postcontrast 3D-FLAIR sequences. In the axial CT image, a dehiscence in the right superior semicircular canal was detected (**C**), which could also be confirmed as a nodular contrast enhancement (array) in 4 h delayed 3D-FLAIR sequences (**D**). About 1/3 of the fluid filling in the RW niche was observed on the axial CT (**E**).

A 77-year-old male was referred to our emergency unit because he first developed a sudden hearing loss on the right side with rotatory vertigo but without tinnitus. On examination, a left spontaneous nystagmus with horizontal and torsional components was noticed. The nystagmus decreased during eye fixation. The PTA was 110 dB on the right side. The VHIT showed a decreased VOR gain of the left posterior (0.26) canals, whereas the other VOR gain was preserved (>0.85). The cervical VEMP was absent on both sides. Moreover, a profound canal paresis on both sides was observed with caloric tests. There were clinical signs assuming a PLF intraoperatively, but the CTP was negative. The contrast enhancement was seen in the right cochlea (Figure 5A) and vestibulum (Figure 5B) on the delayed postcontrast 3D-FLAIR sequences. In the computed tomography, a dehiscence in the right superior semicircular canal was detected, which could also be confirmed as an MR enhancement in that area on the delayed postcontrast 3D-FLAIR sequences (Figure 5D). A hyperintense signal was shown in the left cochlear on the T1 sequence. About 1/3 of the fluid filling in the RW niche was observed in the CT, but in the T2W MRI, none of the fluid fillings were detected.

### 3.6. Case 6

**Figure 6:**
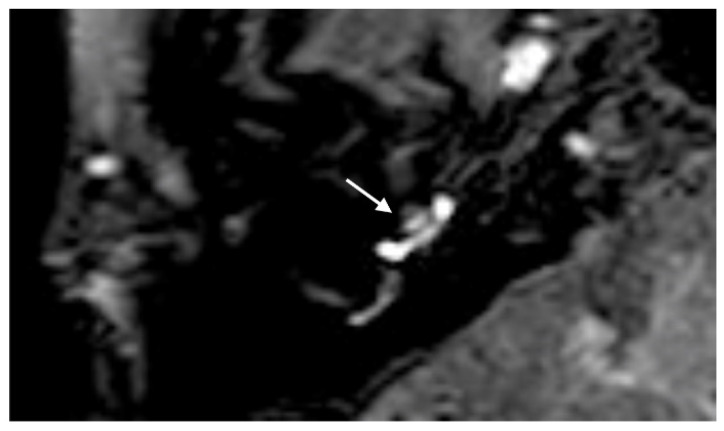
A 66-year-old male with a sudden hearing loss on the right side without vertigo or tinnitus. The contrast enhancement was seen in the right vestibulum and cochlea (array) with axial 4 h delayed 3D-FLAIR sequences.

A 66-year-old male was referred to our emergency unit because he developed a sudden hearing loss on the right side without vertigo or tinnitus. He had already had a sudden hearing loss in history on the ipsilateral side, which was recovered. The audiogram revealed a decreased hearing threshold with a PTA of 92.1 dB on the right side. Intraoperatively, the round niche inspection was interpreted as a PLF, and CTP testing was also positive. The contrast enhancement was seen in the right vestibulum and cochlea with 4 h delayed 3D-FLAIR sequences (Figure 6).

### 3.7. Case 7

**Figure 7:**
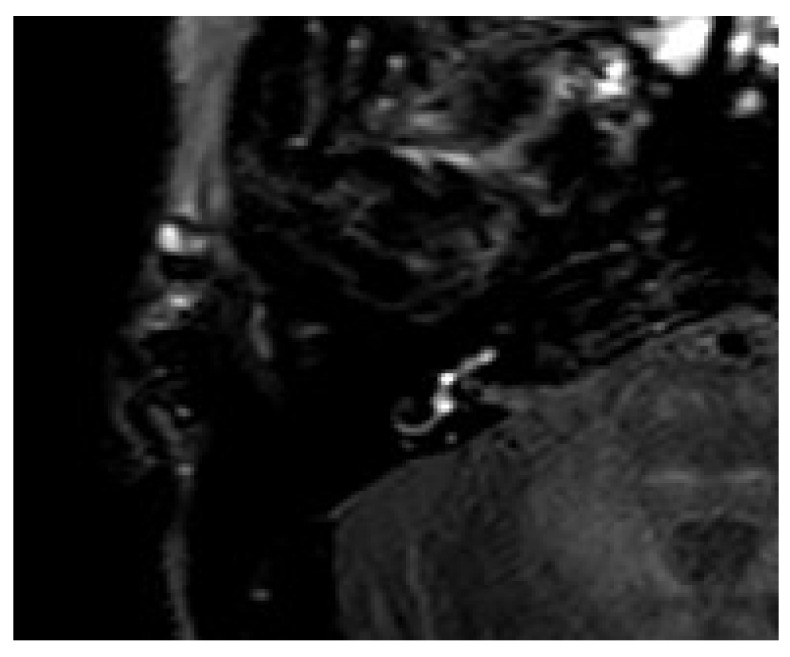
An 84-year-old male presented with an acute hearing loss on the right side with rotatory vertigo. The contrast enhancement was seen in the right vestibulum and basal turn of the cochlea on the 4 h delayed 3D-FLAIR sequences.

An 84-year-old male presented with a sudden hearing loss on the right side with rotatory vertigo. He denied having any other auditory symptoms, such as tinnitus. A left spontaneous nystagmus with horizontal and torsional components was noticed during the examination. The nystagmus decreased during eye fixation. The PTA was 86.4 dB on the right side. The VHIT showed a decreased VOR gain of the left posterior (0.52) canals, whereas the other VOR gain was preserved (>0.85). In the caloric testing, we could observe a canal paresis on the right side. Intraoperatively, the RW niche was wet, but CTP was negative. The contrast enhancement was seen in the right vestibulum on the 4 h delayed 3D-FLAIR sequence (Figure 7).

### 3.8. Case 8

An 85-year-old female presented with sudden hearing loss on the left side and rotatory vertigo. She denied having any other auditory symptoms, such as tinnitus. On examination, a spontaneous nystagmus to the right with horizontal and torsional components was noticed. The nystagmus decreased during eye fixation. The PTA was 110 dB on the left side. A definite MD was previously diagnosed in history.

**Figure 8:**
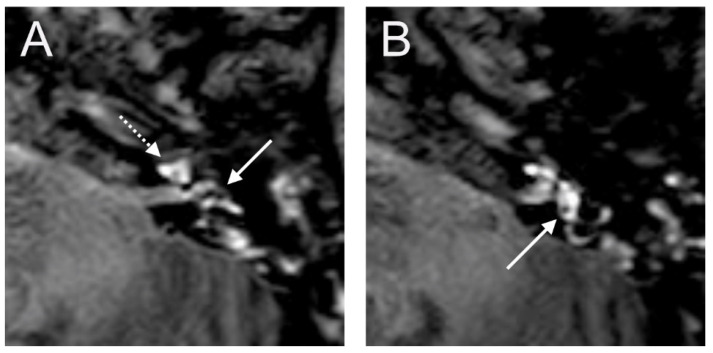
An 85-year-old female presented with sudden hearing loss on the left side with rotatory vertigo. The contrast enhancement was seen in the left cochlea ((**A**) dashed arrow) on the axial 4 h delayed 3D-FLAIR sequences. The inversion of the saccule ((**A**) arrow) to the utricle ((**B**) arrow) area ratio was observed.

The VHIT showed a decreased VOR gain of the left anterior (0.00) and left posterior (0.33) canals, whereas the other VOR gain was preserved (>0.85). Moreover, a profound left canal paresis was observed with caloric tests. Intraoperatively, the round niche was wet, but CTP testing was negative. The contrast enhancement was seen in the left vestibulum and cochlea, and a SURI was observed here on the delayed postcontrast 3D-FLAIR sequences, suggesting vestibular endolymphatic hydrops, grade I (Figure 8). In the CT image, a dehiscence of the superior semicircular canal on the right side could be identified, but these could not be detected with contrast enhancement on the 4 h delayed 3D-FLAIR sequence. On in the T2W image was a hypointense signal intensity observed in the cochlea.

## 4. Discussion

The different underlying pathologies of sudden hearing loss could cause similar audiovestibular symptoms. However, the lack of a definitive diagnostic tool for each cause complicates the therapy process. If a PLF could be pre-operatively detected via MRI, it could ensure the appropriate therapy in time for a PLF related to sudden hearing loss.

In our study, we reported eight patients with sudden hearing loss with or without vertigo and tinnitus. All patients underwent an MRI at different frequencies, including a 4 h delayed intravenous gadolinium-enhanced 3D FLAIR MRI and CTP sample collection performed before sealing the round and oval window during a tympanostomy. We investigated if there was a correlation between the MRI, including a 4 h delayed intravenous gadolinium-enhanced 3D FLAIR MRI, and CTP to diagnose a PLF in patients with acute hearing loss.

MRI scans of all eight patients in our study showed increased contrast enhancement in the area of the vestibulum, cochlea, and fundus of the internal auditory canal at the affected side.

The pattern of those MR enhancements in the vestibulum, cochlea, and fundus of the inner ear canal was various (s. Table 1). Four patients with a positive CTP test, regarded as a PLF, had a contrast enhancement commonly in the vestibulum and cochlea. However, it has been seen not only specifically in MRIs of patients with PLFs (with positive CTP testing) but also in MRIs of the four other patients with negative CTP. Two of these four patients (with negative CTP testing) presented a superior semicircular canal dehiscence and one case of labyrinthitis, and the underlying history of the pathology of the last patient is unclear.

It has already been suggested that the increasing perilymph signal intensity of the 3D-FLAIR sequence is related to the increased permeability of the blood–labyrinth barrier (BLB) in the cochlea. The impairment of the BLB could be shown in various diseases, such as Ménière disease, otosclerosis, and PLF [11,17].

Previously, several studies suggested that an RWS is a sensitive signal on a 4 h delayed 3D FLAIR MRI to detect PLF [11,12,13]. An RWS is characterized as a local high signal around the round window niche on delayed 3D-FLAIR images [18]. In our study, none of the eight patients had a RWS with a PLF (positive CTP). Therefore, we could not find any correlation between an RWS and a PLF. In a study concerning the evaluation of the round window niche on MRI, it was already announced that an enhancement within the round window niche could be an incidental finding without any pathology [19].

A fluid filling in the RW or the OW niches in the CT and T2W MRI was suggested as a PLF-specific signal with 60–80% sensitivity and specificity [13]. In our study, we could observe a fluid filling in the RW niche on the CT image in two cases (case 1 and case 5). In case 1, the mesotympanon and hypotympanon were also filled with fluid. Therefore, it could not be precisely meant that the fluid filling of the RW niche in this case was because of the PLF alone. In the CT of case 5, we could observe a 1/3 fluid filling of the RW niche, but in this case, the CTP was not high for PLFs. On the T2W MRI of both cases, we could not identify any fluid filling in the RW and OW niche. Furthermore, no pneumolabyrinth could be detected in the CT in any of our PLF patients with positive CTP. We assume that microfissure or slow leakage of perilymph in our cases with PLFs could be possibly responsible for a missing positive sign of PLFs in the radiological assessment.

We initially raised the hypothesis that a perilymphatic collapse on delayed 3D-FLAIR might be observed if a PLF exists and the vestibulum lost its perilymph. In lateral comparison in MRIs of all patients, we could not find any significant correlation between PLFs and collapse pattern in the perilymphatic area on delayed 3D-FLAIR MRI. However, two cases are still notable with endolymphatic hydrops detected on delayed 3D-FLAIR MRI in the affected ear (cases 3 and 8). In case 3, the CTP value was corresponded with sure PLF (>60 ng/mL), and in this case, the patient had no audiovestibular symptoms before. The patient in case 8 with pre-existing MD in their history had a CTP value that was not positive for definitive PLFs (CTP = 58.7 ng/mL, intermediate PLFs).

The interaction between perilymphatic and endolymphatic spaces was announced to define the pathomechanismus of the PLF and MD in various studies. Fitzgerald described that the compensatory endolymphatic hydrops happen by the loss of perilymph and also through pressure difference in the peri/endolymphatic spaces [20]. Eliezer et al. presented patients with sudden hearing loss whose delayed 3D-FLAIR MRI showed no visible saccule. Two patients had a surgically confirmed PLF. They hypothesized that an NVS might be caused by a saccular rupture based on a PLF [14]. Goode focused on perilymph hypertension, which could also cause similar symptoms, such as MD [21]. Potter and Conner presented two cases of PLFs who had hydrop-like symptoms after the sealing of the PLF. They suggested that the elevated pressure in the perilymphatic space after the closure of the fistula might be the underlying pathomechanism [22]. Based on those previous studies, we raised a possible explanation for our two cases (cases 3 and 8): compensatory endo/perilymph hydrops are possible through pressure difference in each space. An endolymphatic hydrops could cause perilymphatic hypertension and a PLF, with a leak as a result. However, in case 8m the CTP value was not high enough (CTP = 58.7 ng/mL, intermediate PLF), which means not sure for the PLF. We hypothesize that (1) due to the fine mechanism of an endo/perilymphatic space shift, a PLF occurs accidentally upon the grade of endolymphatic hydrops, as in case 3. (2) In the case of pre-existing MD, as in case 8, the CTP value was not high enough, abd if a slow leak of perilymph already happened, there is barely perilymph existing as MEL performed (for CTP testing) during explorative tympanotomy.

Diagnosis of PLFs is difficult because clinical symptoms of PLFs are variable and non-specific and usually include fluctuating hearing, dizziness with or without vertigo, tinnitus, and ear fullness. Our study could confirm the various symptoms of PLFs. Three of four PLF patients (positive CTP) had a sudden hearing loss in all tone frequencies (cases 1, 3, and 4). The last patient of these four CTP-positive patients suffered from a sudden hearing loss just in the low-frequency range (case 6). All four patients presented no vertigo or ear fullness subjectively. Two out of four patients with PLFs (CTP positive) had tinnitus on the affected side (cases 3 and 4).

The intraoperative sign of a PLF is regarded as another diagnostic method for a PLF, which is defined by the surgeon [2]. Out of seven patients with an intraoperative PLF sign, three could be diagnosed as a PLF with positive CTP. One patient had no intraoperative PLF sign, but CTP test was positive. Even in all four patients without a PLF (negative CTP), a PLF could be surgically confirmed. Other studies have already raised concerns that the intraoperative observation of fluid in the RW or OW niches could be regarded as a PLF false positive because the accumulation of fluid is also possible through CSF, a local anesthetic, or transudates. Through objective CTP testing, we confirm that surgical confirmation of a PLF might be an unclear diagnostic method for a PLF.

The hyperintense signal on the T1W sequence in case 5 and on the CE-T1 sequence in case 6 could not be clearly classified, but Weissman et al. suggested a hemorrhage-caused hyperintense signal on unenhanced MRI in their cases with sudden hearing loss and vertigo [23]. In experimentally produced PLFs by the elevation of cerebrospinal fluid pressure in animal models, an RW rupture and massive bleeding in the scala tympani and cochlear aqueduct were observed [24]. If the hyperintense signal in MRI in our two cases (cases 5 and 6) was caused by hemorrhage, it seems to have variable results because the CTP value of case 6 suggests a PLF, which means that a hemorrhage in the cochlea could be associated with a PLF. But, in case 5, the CTP value was not high for a PLF, which suggests that a hemorrhage could also happen without causing a PLF. Therefore, a hemorrhage sign in the MRI could not verify a PLF.

In light of the studies’ limited number of cases, further studies on a broader scale are necessary for more conclusive results. With our study setting, we could not verify the hearing recovery of patients with a PLF by just surgical treatment through sealing the RW and OW niches because all patients got steroid treatment before. Also, for the same reason, the effect of the time interval between the onset of symptoms to the tympanostomy with RW/OW sealing could not be discussed in our study setting. Finding an appropriate method to diagnose a PLF pre-operatively without surgical access remains unsolved.

## 5. Conclusions

In all patients with sudden hearing loss (>60 dB) independent of the CTP value, a non-specific contrast enhancement in the cochlea and vestibulum could be observed on delayed 3D-FLAIR MRI. The radiological signs for PLFs, such as RWS, pneumolabyrinth, or RW fluid signal, could not be detected in our cases with PLFs. Using multimodal diagnostic measures, such as CTP testing and various MRI sequences, no correlation could be found in patients with PLF in our study.

## Figures and Tables

**Table 1:** The results of clinical tests and a CTP test in patients with sudden hearing loss (sure for PLF if CTP > 60 ng/mL).

Case	Sex	Age, yr	Affected Side	From Onset of Symptom Until Hospital Admission (Day)	PTA (First Examination)	PTA (3 Weeks Post-OP)	Intraoperative Sign of a Fistula	CTP (ng/mL)	Vertigo-Symptom	Tinnitus or Ear Fullness	VOR (Gain)	Caloric	Pathology
1	M	60	Right	1	110	110	Wet	**880.0**	No	-	Right anterior (0.00)	Deficit right	PLF
2	F	38	Left	8	110	110	Wet	47.3	Yes	-	Norm	Deficit left	Labyrinthitis
3	M	48	Right	10	110	77.8	Wet	**99.5**	No	Tinnitus	-	-	PLF/EH
4	M	86	Left	21	110	41.4	Dry	**84.0**	No	Tinnitus	-	-	PLF
5	M	77	Right	7	110	82.1	Wet	21.6	No	Tinnitus	Left posterior (0.26)	Deficit both	SSCD
6	M	66	Right	0	92.1	75.7	Wet	**74.6**	No	-	-	-	PLF
7	M	84	Right	0	86.4	57.1	Wet	13.3	Yes	-	Left posterior (0.52)	Deficit right	Acute audiovestibular dysfunction
8	F	85	Left	0	110	110	Wet	58.7	Yes	-	Left anterior (0.00) and left posterior (0.33)	-	EH/SSCD

**Table 2:** MRI analysis in patients with sudden hearing loss.

		4 h Delayed Intravenous Gadolinium-Enhanced 3D FLAIR MRI			MRI				Computed Tomography		
Case	Affected Side	MR Enhancement	Round Window Sign	Other Charateristics (EH? SSCD?)	T1	T1 with CM	T2	Presence of Fluid in the RW Niche in T2?	RW Fluid Filling?	Air	Dislocation or Intravestibular Depression of the Footplate?
1	Right	Cochlea basal turn, vestibulum right	No	No	-	-	-	Not sure	3/3 of RW (as well as meso/hypotympanon; filled)	not	No
2	Left	Cochlea, vestibulum, fundus left	No	No	Hyperintense	Hyperintense	Hypointense	No	No	No	No
3	Right	Vestibulum, fundus, cochlea right	No	Vestibular EH right (grade I)	-	-	-	No	No	No	No
4	Left	Cochlea basal turn, vestibulum left	No	No	-	-	-	No	No	No	No
5	Right	Cochlea, vestibulum, fundus right	No	SSCD right	Hyperintense	-	-	No	1/3	No	No
6	Right	Vestibulum, fundus, cochlea right	No	No	-	Slightly hyperintense	-	No	No	No	Not sure
7	Right	Vestibulum right	No	No	-	-	-	No	No	No	No
8	Left	Cochlea, vestibulum left	No	Vestibular EH left (grade I), SSCD left	-	-	-	No	No	No	No

## Data Availability

The original contributions presented in the study are included in the article, further inquiries can be directed to the corresponding author.
